# Recent epidemiologic trends of HAM/TSP in Japan

**DOI:** 10.1186/1742-4690-8-S1-A77

**Published:** 2011-06-06

**Authors:** Toshio Matsuzaki, Tomoko Kodama, Ryuji Kubota, Shuji Izumo

**Affiliations:** 1 Center for Chronic Viral Diseases, Kagoshima University, Kagoshima 890-8544, Japan; 2 National Institute of Public Health, Saitama 351-0197, Japan

## 

Recently a survey of HTLV-1 carrier in Japan has been done based on the positivity of Red Cross blood donors, and demonstrated that HTLV-1 carriers are not reduced and diffused from south-western endemic areas to central metropolitan cities in Japan. The last nation-wide survey of HAM/TSP has been conducted in 1994 and reported 1103 cases of definite HAM/TSP in Japan. In order to investigate recent trends of HAM/TSP in Japan we conducted a nation-wide search of HAM/TSP patients who have attended 829 neurology clinics in recent two years, 2007 and 2008. Altogether 277 clinics (33.5%) responded and 790 cases of HAM/TSP were registered. Simultaneously registered amyotrophic lateral sclerosis (ALS) patients were 1,002 cases, and the number of HAM/TSP in Japan was estimated to be less than 3,600 when compared to that of ALS (4,900). Male to female ratio was 1:2.5 and mean age of onset was 49.4 years old. More than 30 patients per year were found to have an onset of the disease in these 10 years. Concerning the distribution, 52% of patients are living in Kyushu, whereas, 15.4% are living in the area around Tokyo and 15.9% around Osaka. The ratio of patients living in such areas of central Japan was increased when compared those with onset before 1995 and those after then. In conclusion, the number of patients with HAM/TSP is not reduced and more patients are found in the metropolitan areas.

